# Collaborating with Communities and Higher Education to Address the Health-care Needs of Individuals with Disabilities in Ecuador

**DOI:** 10.3389/fpubh.2017.00091

**Published:** 2017-04-21

**Authors:** Donna J. Cech, Zully J. Alvarado

**Affiliations:** ^1^Department of Physical Therapy, Midwestern University, Downers Grove, IL, USA; ^2^Causes for Change International, Chicago, IL, USA

**Keywords:** community-based rehabilitation, individuals with disabilities, health care, international collaborations, global health

## Abstract

Individuals with disabilities experience inequities in access to health care, education, employment, and social inclusion. Causes for Change International (CCI), a non-governmental Organization (NGO), using a community-based rehabilitation approach has worked for 20 years to build self-sufficiency, improve health-care services, and education for women, children, and persons with disabilities in Ecuador. CCI initially addressed health; advocacy for individuals with disabilities; and promoted educational opportunities for children with disabilities, starting in one rural community. CCI’s outreach has expanded through Ecuador’s coastal provinces, Andean provinces, and Galapagos Islands. CCI also focused on local health-care workforce development, developing employment skills for individuals with disabilities and social inclusion for this population. CCI collaborated with local organizations, government, and universities to provide resources, managed by local leadership. Key program elements of the CCI approach include (1) develop trust between CCI, local communities, local agencies, and government; (2) empower local groups to assume leadership and sustain programs; (3) support communities and groups invested in developing self-sufficiency; and (4) strengthen collaborations and partnerships between local and international organizations, universities, and government agencies. Key lessons learned by CCI are to be supportive of cultural differences; understand that limited financial and material resources may limit the program development; recognize that it is difficult not to foster dependent relationships with communities and appreciate the importance of working with and within the host country’s governmental systems. CCI is expanding its service base to other regions of Ecuador and is focusing on development of the Ecuadorian health-care workforce and social inclusion opportunities for individuals with disability. The efforts of a small NGO have helped build community self-sufficiency in meeting the health care and rehabilitation needs of all Ecuadorian citizens and a greater awareness of the abilities and potential contributions of individuals with disabilities.

## Introduction

Global inequality in access to the health-care resources exists across the globe. Health care needs are the greatest in developing countries and in countries with significant proportions of their population living in rural areas. Health-care resources are difficult to access for indigenous populations and individuals with disabilities. Individuals with disabilities also experience inequities in other aspects of life such as education, employment, and social inclusion. Health is defined as a state of complete physical, mental, and social well-being and not merely the absence of disease or infirmity (1). To most positively impact health of individuals with disabilities, all three of these domains of health must be addressed. Individuals with disabilities, in developing countries, face challenges both related to living in their country and with their disability. Access to health is impacted by economics, transportation challenges, decreased accessibility of buildings, limited understanding by health-care workers regarding health-care needs, and limited knowledge of individuals with disabilities regarding their rights and needs related to health care (2). Global efforts have focused on improving health and well-being of individuals in developing countries and often have involved multinational collaborations to meet the needs of all citizens of the world.

The community-based rehabilitation (CBR) approach was introduced and adopted by the World Health Organization more than 25 years ago, as a way to support and empower individuals with disabilities where access to support systems and rehabilitation might be limited. CBR programming strives to enhance health care, education, social inclusion, and employment opportunities, leading to an improved quality of life through collaborative efforts of multiple partners (3). The approach strives to help individuals with disabilities become contributing members of their communities. The five key components of CBR, called the CBR matrix, are health, education, livelihood, social inclusion, and empowerment for individuals with disabilities (3, 4).

Community-based rehabilitation programs are usually initiated by governments or by non-governmental organizations (NGOs). NGOs are not-for-profit, private organizations that have assumed a greater role in global health and development since the 1980s, providing private support to reduce poverty, improved health care, and assistance in supporting development, within low- to middle-income countries (5). Many NGOs focus on bringing services to communities with limited services and underserved segments of a society. Within the CBR model, stakeholders in local communities develop partnerships with the NGO or other umbrella group to bring quality services into the community, close to people’s homes. Local partners take an active role in developing and supporting activities. Individual stakeholders and their families, community members, local agencies, and local government then collaborate with the outside NGO or governmental agency, ultimately assuming management of the CBR program. Through these partnerships, the umbrella agency, university, or NGO can provide mentorship for local partners. The mentorship and involvement of local stakeholders increases the sustainability of the CBR program (6, 7).

Important elements of CBR include acknowledgment and respect of local customs, establishment of community partnerships, and involving individuals with disabilities and their families into decision-making (3, 8). By respecting local customs and knowledge, CBR programs are more likely to become part of the community context. Working within the cultural framework helps the community see the contribution and needs of individuals with disabilities. Individuals with disabilities are empowered to become participants and valued members of the community. When community partners are active in the development and implementation of CBR initiatives, program sustainability is enhanced (9). When individuals with disabilities and their families are involved in the decision-making, they become more active community members and gain leadership skills. Within the collaborations, it is important to recognize the goals of the local community and that all collaborators are equal partners in best meeting the needs of the community.

Ecuador, a geographically diverse country, has a population of approximately 16 million people, including several indigenous groups. Almost two-thirds of the people live in urban areas and one-third in rural areas (10). Until recently, most health-care services were available only in the largest cities. Over the past decade, the government has built infrastructure including hospitals and clinics. In the 1990s, the WHO identified Ecuador as having a high global burden of disease, with high child and adult mortality (11). From 1990 to 2015, significant improvements were made in lowering infant, child, and maternal mortality; improving sanitation and water supply; and reducing incidence of diseases such as malaria (9). In 2007, the Ecuadorian government worked to determine the number and needs of individuals with disability in the country. The government then began providing financial assistance for individuals with disabilities. Ecuador ratified the United Nations Convention on Rights of Persons with Disabilities in 2008 (12). Services, opportunities for education, and jobs for individuals with disabilities have increased, supporting inclusion of these citizens within their communities. The Ecuadorian government has also been instrumental in supporting training of the elementary education teachers and a highly skilled health-care workforce.

Causes for Change International (CCI), a NGO, was founded 20 years ago to assist the people of Ecuador build self-sufficiency and improve services related to health, education, and economic self-sufficiency for women, children, and persons with disabilities. CCI took a grassroots approach, working with local citizens in the poorest communities and helping build health care and community-based services. Early focus was on domestic violence, dentistry, and services to increase independence of individuals with disabilities. Through outreach, training, and partnerships with local organizations (elementary schools, community service groups, NGOs), government (local and provincial), community health centers and universities, CCI has built stronger communities, managed by local leadership. CCI now has collaborations with local and provincial government in several provinces, the Ministry of Inclusion, and public University system.

Causes for Change International educated the people who needed services unavailable in small, rural communities. As the citizens learned about improved health and self-sufficiency, they influenced and educated local government and organizations to gain support for projects. Involving local citizens in identification of solutions developed needed local leadership to implement and sustain services. Five dental clinics were set up in small towns, with CCI assisting in acquisition of necessary equipment and supporting young Ecuadorian dentists to practice in rural areas. Local involvement resulted in expansion of these clinics to include health care and rehabilitation services. Local health-care workforce development was targeted through continuing education courses, taught by CCI volunteers. A school for children with disabilities was developed, and professional education programs in social work and physical therapy are being developed within a public university. The purpose of this case report is to describe the development of a sustained collaboration between CCI and Ecuadorian partners in improving the health, social inclusion, and quality of life for individuals with disabilities in Ecuador.

## Context

**Figure 1:**
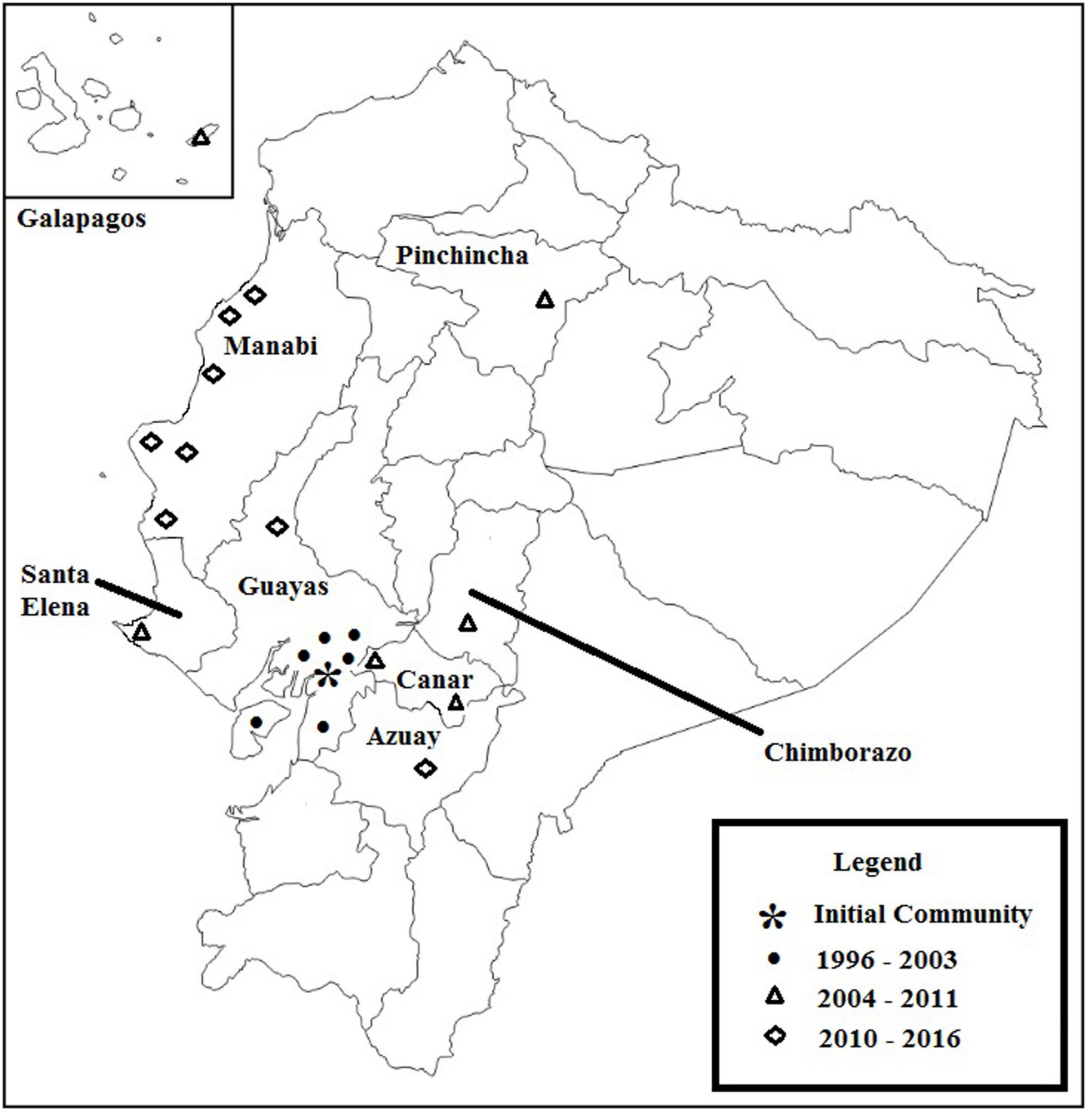
**Causes for change international involvement in Ecuador**.

Causes for Change International’s initial outreach was to an economically challenged, rural community near the cities of Guayaquil and Milagro, Ecuador. These cities are part of Guayas province, but, in 1996, were not easily accessible by road from the community. This region, commonly referred to as the “coastal region” supports banana, plantain, cacao, and sugar cane production. Guayaquil is also a major shipping port. CCI expanded outreach through Guayas and eventually expanded through the “coastal region” to communities within Manabi province and other regions within Ecuador (see Figure 1).

Local citizens, supported by CCI, approached local businesses, agencies, and government to invest and improve the community. The University of Milagro (UNEMI), a regional public university, became an early partner in these efforts. CCI worked with local leaders to establish locally run clinics and disability advocacy programs. These services were made available to individuals with disabilities, in the local community. CCI also became active in Azuay, Cañar, Chimbarazo, Pichincha, and Santa Elena provinces and the Galapagos Islands.

### Key Program Elements

The CCI approach has been centered on elements of: (1) developing trust between communities, CCI, local/regional services, and all CCI partners; (2) empowering local groups to develop, lead, and sustain programs; (3) focus on communities and groups that are invested in developing self-sufficiency; and (4) facilitating development of strong community partnerships. Development of trust has been reciprocal. Local communities needed to trust that CCI would be a consistent resource, not just a one-time event. CCI also needed to trust that the community would follow through and assume responsibility for initiatives. In addition, CCI needed to foster trust and respect between local communities, international volunteer teams, Ecuadorian service agencies, and providers. Local professionals and international volunteers learned from each other, valuing each other’s expertise. This professional interaction established local professionals as valued partners, reinforcing their ability to carry out community initiatives. When developing trust, it is also important to honor the cultures of the citizens and communities. Beliefs and cultures of members of indigenous communities, rural communities, and urban communities vary. In empowering local groups, CCI works with communities who have identified a need and asked for assistance. CCI mentors local community leaders to assume management of the efforts. CCI asks local hosts to assist with expenses related to local travel, housing, and meal costs for international volunteer teams, demonstrating a commitment by the community and building self-sufficiency of local groups. Sustainability is also strengthened by building local partnerships. For example, a school for children with disabilities was linked with a University to provide professional internships and student teaching opportunities. To meet a need for wheelchairs, CCI partnered with Free Wheelchair Mission involved engineering students from a state university to assemble all wheelchairs and helped chapters of the Lion’s Club of Ecuador distribute the wheelchairs throughout the country. A local organization has now assumed responsibility of the relationship with the international organization regarding acquisition of additional wheelchairs.

Causes for Change International’s activities have paralleled the components of the CBR matrix, focusing initially and primarily on health services and elementary education. These health services have included prevention efforts in improving sanitation; provision of needed dental, medical, and rehabilitation services; and providing access to assistive devices. Currently, CCI activities support development of a well-qualified local professional workforce. Consistent efforts have also focused on the empowerment component, through advocacy, communication, and support of organizations addressing the needs of individuals with disabilities. CCI has also participated in job skill development activities, supported employment of individuals with disability in the shipping and shrimp industries, and collaborated with local governmental service agencies to provide for the needs of individuals with disabilities. A timeline describing CCI initiatives and partnerships (Table 1) demonstrates the evolution of CCI activities and involvement, starting in one community and expanding across Ecuador.

**Table 1:** **Causes for Change International (CCI) community-based rehabilitation activities in Ecuador**.

	1996–2003	2004–2011	2012–2016
Health: general health and hygiene	Community trash collection with local businesses asked for donations of gloves, brooms, etc.	Partnered with CCI-Ecuador to bring clean drinking water to communities where it had not been available	Earthquake relief/response related to housing and health care
	Hygiene (hand washing and dental health) curriculum in local schools and orphanage		
	Domestic violence awareness		

Health: provision of medical, dental, etc. services	CCI volunteers provided medical, dental, audiology services	CCI volunteers and local professionals provided medical and dental services to 7,000+ children, optometry services to 400 children	Supported development of University training programs for health-care professionals in social work, occupational therapy, and physical therapy
	Donation of hearing aids for 500 children	Planned and implemented opening of 5 Ecuadorian dental clinics, donating dental chairs from USA	Partnered with local non-governmental organization to provide medical, dental, and therapy services in Alausi, provide services in Canar
	Establishment of partnership with local hospitals to provide cardiac surgery, craniofacial surgery, burn care to local children		

Social inclusion and quality of life	Formed relationships with (1) schools in Guayaquil for children with special needs; (2) program for children with Down syndrome	Donated assistive devices (walkers, crutches, 500+ wheelchairs)	Partnered with Free Wheelchair Mission -80 wheelchairs to Ecuador
	Staff training at rehabilitation lefts	Disability awareness, inclusion, and universal design training to special education, primary school teachers, education, and medical students; University of Milagro (UNEMI) and Milagro local government	Accessibility and Universal Design Conference in Guayaquil
			Needs assessment of disability community in the Galapagos
	Disability awareness “Disability March” in Manta	Disability conference for governmental agencies and NGOs in Ecuador	OT and PT Conference on Management of the Child with Cerebral Palsy
		Training at UNEMI, Naval and Military Hospitals	Physical therapy consult/training for families of children with disabilities scheduled for orthopedic surgery (sponsored by CCI—Manabi)
		Met with senior staff of Vice-President of Ecuador; CCI invited to work with government on making Ecuador accessible	Develop national assistive device lending library, Bucay
		Begin development of Exploration Institute	Presentations to families of children with Down Syndrome and professionals working with them in Quito
			Support (training, medical/dental/therapy services) to community built for individuals with disabilities in Milagro

Leadership development	Trained women in 26 km, local schools, and organizations	Causas para el Cambio—Ecuador established with its own volunteer board	Ecuadorian delegations and individual leaders visited USA sites to learn about access, professional education programs, and nutrition
	Incorporated CCI component, CCI-Ecuador with local leaders	Provided support and mentoring to orphanage in Guayaquil to open bakery and help girls develop bread and pastry baking skills	Supported existing CCI-Ecuador leaders and communities with Earthquake relief
		Hosted Ecuadorian delegations in USA for leadership training, disability awareness	

#### The Early Years from 1996 to 2003—Identifying Needs and Building Local Leadership

##### Health Initiatives

Original CCI initiatives focused on serving populations in small, rural communities. Leadership training of local community members and education related to health and hygiene, domestic violence, and disability advocacy were provided. Local citizens solicited assistance from local businesses and local government, increasing awareness of local needs and the issues important to the community. General medical services in the initial community served were not readily available in the community. CCI volunteers (physicians, nurses, dentists, audiologists, educators) traveled to Ecuador 1–2 times per year to provide services and help link the community with local hospitals in Guayaquil and Milagro. CCI volunteers (environmental engineer, teacher) also stayed for longer periods of time to support the local leadership in sanitation efforts and develop health, hygiene, and personal development skills at a local orphanage and schools.

##### Social Inclusion and Quality of Life Initiatives

Causes for Change International formed relationships with schools in Guayaquil for children with special needs, provided staff training at rehabilitation centers and assisted the local disability community in organization of a “Disabilities March” in Manta. Relationships were also established with a program for children with Down syndrome and continued support for a local orphanage was provided, creating a playroom with educational toys and a library.

Another main focus of CCI was development of a local leadership team. In 2001, a 6-week leadership training course was sponsored for individuals, personnel from local schools, and local organizations. By 2003, these local leaders urged CCI to assist them in incorporating an Ecuadorian division of CCI and Causas para el Cambio—Ecuador was incorporated.

#### The Middle Years, 2004–2011—Mentoring and Supporting Local Initiatives and Building Expanded Partnerships

##### Health Initiatives

Within this time period, Ecuadorian partners began initiating events and requesting support from CCI. CCI continued to bring volunteer providers (physicians, nurses, therapists, dentists, optometrists) to Ecuador every year, to meet basic needs of local populations. On one visit, CCI volunteers worked with a boy with bilateral lower extremity amputations, who had no means of mobility other than scooting on the ground. CCI leadership worked with provincial officials in Guayas and the local mayor to identify services for the child. A CCI volunteer brought a hand-propelled tricycle, donated by AMBUCS organization in the USA. CCI also initiated fund raising and made contact with a prosthetist in Quito so that the boy could receive prosthetic limbs. CCI brought donated equipment to help support the opening and operation of Ecuadorian run dental and rehabilitation clinics, bringing these health-care services to individuals with disabilities in their communities. Causas para el Cambio—Ecuador also implemented a new clean water initiative to help bring clean drinking water to areas where it was unavailable, improving health of the communities.

##### Social Inclusion and Quality of Life Initiatives

Causes for Change International continued to focus on the needs of individuals with disabilities and shipped containers of crutches, walkers, and wheelchairs to Ecuador. CCI met with the Ecuadorian government and was invited to help make Ecuador accessible for persons with disabilities. CCI participated in a disability conference for governmental agencies and NGO in Ecuador and provided training on disability awareness, inclusion of persons with disabilities, and universal design to education and medical personnel. CCI volunteers included physicians, architects specializing in accessibility, and disability advocates. Consultation was provided to the city of Milagro and UNEMI regarding building of accessible ramps on campus and in city parks. The idea of developing an “Exploration Institute” for children and youth with disability was also introduced with local government and leadership in Guayas province. This institute would provide an opportunity for children and their families to enhance the child’s strengths and abilities while exploring nature and the environment.

Local leadership development continued with Causas para el Cambio—Ecuador establishing a volunteer board. CCI continued to provide mentorship and support to the Ecuadorian NGO, as it also developed branch organizations in Manabi province. The orphanage in Guayaquil, an early CCI partner, requested assistance in providing job training to the girls living in the orphanage so that they would have the skills to become self-sufficient. CCI provided them support and mentoring to open a bakery business and develop bread and pastry baking skills. Partnerships were expanded to include: Ecuadorian and USA universities; Ecuadorian agencies and service organizations, service organizations in the USA, and several local government units (Table 2). In this middle period, CCI continued with a focus on the health and empowerment components of the CBR matrix, as well as with initiatives that addressed livelihood and social CBR components.

**Table 2:** **Partnerships fostered and developed with CCI**.

	Ecuador	USA and International
Local agencies/NGO	Causas para el Cambio-Ecuador	CCI
	□ Manabi	Starkey Foundation (hearing aids)
	Instituto Perpetuo Socorro (orphanage)	Free Wheelchair Mission
	AVINNFA—school for children with disabilities	Manaakii Foundation (mobile dental clinic funding)
	Voluntad de Dios, Milagro, EC (accessible housing community)	Nuestros Pequeños Hermanos Internacional
	Lions Clubs—Ecuador (35 chapters)	Encuentro Medico de Alausi
	Foundation SOMAS	Rotary Clubs of Cicero and Chicago, IL, USA
	Galapagos fire department	
	Rotary Club of Guayaquil	

Local government	Mayoral offices in Manta, Jama, Ayampe, Canar (city of), Milagro, Bucay, Porto Viejo, Porto Lopez, Montichristi, Naranjal, Jipijapa, Bahia	Town of Cicero, IL, USA

Provincial government	Office of Prefectura—Manabi, Guayas	
National government	Office of the Vice-President re-accessibility and needs of individuals with disability	
	Ministry of Inclusion—MIES	

Universities	UNEM	University of Illinois at Chicago
	University of Guayaquil	Dominican University, IL, USA
	Technical University of Manabi	Midwestern University, IL, USA
	University of Agronomy, Naranjal, EC	

#### The Most Recent Years, 2012–2016: Supporting Local Leadership and Initiatives, Building Professional Workforce

##### Health Initiatives

Today, CCI’s reach has expanded to provide education and support to provincial governments, local hospitals, universities, and organizations throughout coastal and highland areas. Although basic health services continue to be provided, new initiatives focus on helping to build health-care workforce capacity and services for individuals with disabilities. Workshops have been developed to provide advanced training to Ecuadorian health-care providers. Long-term partnerships have been established between universities in the USA (Dominican University and Midwestern University) and UNEMI to develop university-based training programs in social work, physical, and occupational therapy. When developing the social work program, CCI linked UNEMI and Dominican University to an elementary school for children with disabilities, increasing services for the students and their families. UNEMI has also integrated student internships and student teaching at the school, building the local workforce available to work with this population. Social work students from Dominican University have completed internships at the school and in Milagro, providing services within the community. These partnerships have also included faculty exchanges, visits to USA universities by Ecuadorian university administrators and faculty, and curriculum development.

In Spring, 2016, Causas para el Cambio-Manabi was supported by CCI in recovery efforts after a strong earthquake. CCI and Causas para el Cambio leadership began monitoring local needs immediately after the earthquake and CCI volunteers have assisted in building temporary shelters, as well as distribution of food and medication.

##### Social Inclusion and Quality of Life Initiatives

Collaborative international and local efforts have successfully provided wheelchairs, which were distributed throughout Ecuador. Partners included Rotary clubs (in the USA and Ecuador), Lion’s Club-Ecuador, and the UNEMI. Another local service agency has assumed responsibility for further acquisition of donated wheelchairs. CCI is assisting in the development of a national assistive technology lending library, acquiring a variety of equipment and developing policies and procedures to support the long-term viability of this library. CCI also participated in the Accessibility and Universal Design Conference in Guayaquil, providing volunteer faculty including architects specializing in accessible design, occupational therapists, and disabilities advocates. CCI has hosted a delegation of Ecuadorian community leaders to visit Chicago, where they were able to learn more about providing access for individuals with disabilities.

Causas para el Cambio-Manabi has asked CCI for support in initiating new economic opportunities along the coast that would provide social inclusion in sports, leisure, and recreation activities for individuals with disabilities and build tourism. CCI and Causas para el Cambio began working with Olas para Todos (Waves for All) and the Office of Tourism in developing a surfing program, which would (1) enhance the abilities of children and adults with disabilities to encourage participation, (2) provide internship opportunities for university students majoring in the health professions to work in this specialized field, and (3) create programs to increase tourism in the area. These most recent initiatives continue to support the CBR matrix components of health, education, social inclusion, and empowerment of individuals with disabilities.

As local leadership assumes primary responsibility for efforts in Guayas and Manabi provinces, CCI has expanded the geographic scope of its CBR efforts in Ecuador. A needs assessment of the disability community in the San Cristobal Island, Galapagos was completed by USA volunteers. Presentations were made to service organizations in Quito serving children with Down syndrome and their families and children with cerebral palsy. Health services and training were provided in highland areas of Alausi and Cañar, and a formal partnership with the local government of Cañar has been established.

## Discussion

Over the past 20 years, CCI has noted improved awareness of the needs, abilities, and potential contributions of people with disabilities in Ecuador, as well as availability of services. Letters of agreement and collaborations were developed between CCI and local communities, provinces, local agencies, and a public university. CCI is discussing a collaboration with the Ministry of Inclusion of Ecuador, partnering with Universities to develop health-care education programs and provide continuing education resources for practicing professionals. CCI’s CBR initiatives have evolved from partnering with one small, poor community and have expanded across Ecuador.

Local leadership development has been demonstrated by the formation of Causas para el Cambio—Ecuador, with a provincial chapter in Manabi, and identification of local representatives in several communities. Local dental and health clinics have been developed and continue to operate in several communities. Not all efforts have been sustained. In one community, training and donation of school supplies were part of initial interactions. The community requested help in developing a park, but after several visits by CCI, the local community did not make progress in this planning and, instead, requested continuing financial assistance from CCI. Because the local community did not appear to be able to assume responsibility for development of the initiative, CCI was unable to continue to work with the group. Changes in local government have also led to dissolution of programs, despite community support for them. In a recent election cycle, new local government officials closed one of the dental and rehabilitation clinics, claiming the newly refurbished, accessible space as the local government office space.

### Practical Implications and Key Lessons Learned

CCI’s 20-year experience in Ecuador has demonstrated that individuals with disability have a desire to be active in their communities. For this to occur, CCI has learned that community participation starts within the family. Family acceptance of the family member with disability is important, but often limited by family belief systems and embarrassment. Families need support to appreciate the role and value of the family member within the family unit and community. As families feel more empowered to bring individuals with disability into community activities, community beliefs regarding the value of the individual to the community are also enhanced. Community education efforts assist in the inclusion of individuals with disabilities in the community. Increased community engagement and understanding of the needs and contributions of individuals with disability then lead to enhanced local government support.

The CCI experience has also shown that local leadership, with support and mentoring, can develop and implement community services. Today, local leaders are successfully assessing community needs and mobilizing resources to provide needed services.

In thinking about the programs that have been sustained and those that have not achieved self-sufficiency, CCI has learned some key lessons. An important lesson learned was that foreign NGO’s may not understand local customs, culture, and resources, bringing unrealistic expectations of local communities (6). It is very important to be supportive of cultural differences in management of time and organizational structures. Foreign volunteers need to be patient as timelines are often extended. It may also be difficult to identify and work with appropriate personnel within agencies and government. The reliability of systems of communication may be an issue. Although frustrating for many foreign volunteers, these factors reflect the culture within which they are volunteering.

Non-governmental organizations and volunteers must understand that limited financial, human and material resources may limit the program development. Although many partners may be willing, it is difficult to connect with professional support to move a project forward. For example, CCI volunteers were unable to find local surveyors, when trying to select land for the Exploration Institute, resulting in postponing the project. CCI had also supported land use and flora and fauna studies in another community, but as that community began to appreciate the benefits of increased tourism and the value of that land, it became unavailable for the Institute. CCI has also learned that a sufficient number of local, well-trained professionals to staff and volunteer at the Exploration Institute may not be available. Until a larger local workforce can be developed, it may be difficult to implement the project. These experiences have led to a modification of the original vision for the Exploration Institute, to better meet community needs. It may be possible to achieve the original goal of the Exploration Institute by supporting multiple local efforts to increase social inclusion opportunities for individuals with disabilities, while allowing them to enhance their strengths and abilities. One example of a local program that has the potential to foster economic development and tourism in the community, while providing opportunities for individuals with disabilities in the surfing program being developed in Santa Elena.

Another important lesson has been to recognize the importance of working with and within the host country’s governmental systems. Understanding the local, regional, and national governmental system assists in helping individuals with disabilities access resources. Positive collaborative relationships with governmental agencies and officials also support effective utilization of NGO and local resources. When working with the government on national and local initiatives, the NGO can be supportive and improve effectiveness in meeting the needs of the citizens, but must also be clear that they do not support any specific political party. The NGO should clearly communicate that there is no political or government affiliation, which is important for establishing and maintaining trust with the local community. Both governmental and community CBR efforts are important. Programs sponsored by government may have more resources and reach a broader area, making them more sustainable. Community-run programs may be more effective in difficult (rural or indigenous settings) and invoke greater community participation and ownership. For these reasons, it is important to support collaboration and partnerships between the government and community groups (3).

Finally, it is very difficult to avoid dependent relationships with communities. In situations where resources have been limited, local communities may see free services as a new resource. It may be difficult to help the community appreciate that developing the skills to be self-sustaining is the worthwhile outcome. Local leadership may also desire to maintain a dependency upon the NGO partner. This may be related to local customs and beliefs regarding advocacy and leadership, or may reflect poor self-assurance that the local community can proceed without significant support. If local leadership cannot be mobilized, it may be better for the NGO to move support to another community to maximize potential for developing local self-sufficiency. CCI actively tries to provide support only in the early stages of project implementation. For most projects, the community leaders are very effective in helping the programs thrive.

### Conceptual and Methodological Constraints

Non-governmental Organizations should be cautious about overextending themselves. As CCI became more well known, multiple requests were made for assistance. After collaborating with international and local health-care resources to provide services to a child with severe burns, CCI found itself partnering with local firefighting groups to help them obtain equipment and training. While this does support the health and safety of citizens in the community, these initiatives did significantly expand the CCI scope. Other countries also sought assistance. It was difficult to maintain original focus of CCI, requiring CCI leadership to review the mission and purpose of the organization.

An NGO needs to develop its infrastructure to ensure sustainability. CCI’s structure now includes CCI and Causas para el Cambio-Ecuador, with international and Ecuadorian volunteer corps. Over the past 20 years, CCI has built a substantial Ecuadorian leadership base. Initially, significant efforts were made to help individuals appreciate the concept of volunteerism and the satisfaction and personal growth that comes from being a volunteer. It was often felt that foreign volunteers were wealthy and that was why they could volunteer. Local volunteers now give of themselves, not because they are wealthy, but because they believe that volunteerism is a good way to support their country and humanity. Ecuadorian volunteers are now mentoring and supporting additional volunteers.

The growth and success of CCI’s Ecuadorian initiatives has also brought challenges related to the level of volunteer expertise needed to meet the community’s needs. As CCI has seen local communities become more self-sufficient in meeting basic health care and educational needs of individuals with disabilities, the CCI leadership has needed to reach out to volunteers with different types of expertise to meet the challenges of developing community accessibility, workforce development, and national advocacy.

Causes for Change International has never focused on financial growth of the organization. Trips to Ecuador are self-funded by the international volunteer team and economical travel plans are maintained. Even with this, CCI has had to build a small fund-raising base to cover operational expenses such as phone bills, Internet costs, web page development, local travel, and board meetings. This type of fund raising takes significant time and effort, but is very necessary.

## Conclusion

During the 20 years, CCI has been working within Ecuador, the national government has greatly enhanced the country’s infrastructure related to transportation and health care, making access to services and mobility through all regions of Ecuador possible. As monitored by the World Health Organization, improved health, economic, and health-care workforce status has been seen from the 1990s to present (8). CCI is fortunate to have been working in Ecuador at this time and has hopefully helped services reach the rural, indigenous, and poor communities of Ecuador. In 2017 and beyond, CCI hopes to contribute to implementation of the adaptive equipment lending library for the country, development of the Exploration Institute, expansion of efforts to build disability awareness and inclusion of individuals with disabilities in community activities, recreation, and leisure activities. CCI would like to work with local leaders in the Galapagos Islands and other regions of Ecuador to better meet the needs of individuals with disabilities. In addition, CCI hopes to partner with agencies and universities to facilitate advanced continuing education courses, develop professional education programs, and to assist in local faculty development.

The methodology and approach that CCI has followed when working with communities meets the ethical considerations of a CBR approach, which is important to support fairness and social justice in providing needed services to people with disabilities (8). The Ecuadorian government also supports a CBR approach to best serve the needs of the citizens of Ecuador and is requesting all health-care provider education programs to include a CBR experience. Recommendations for strategies to help meet the needs of individuals with disabilities and their families within their communities are summarized in Table 3.

**Table 3:** **Recommendations to meet the needs of individuals with disabilities and their families within their communities**.

	Recommendations
Health	Initially, need to evaluate needs of communities and individuals with disability within the community Provide individual patient care and screenings with volunteer providers Link individuals with special needs with local and regional resources Meet needs of individuals Build awareness among local resources related to needs of individuals with disabilities Support local health-care workforce development Continuing education programs Professional preparation programs in university settings
Social inclusion	Empowerment of individuals with disabilities Build positive self-concept Build confidence related to abilities for individual, family, and community participation Work with families if individuals with disabilities Address issues related to feelings of guilt, cultural beliefs, and values Build awareness of the role and value of the individual with disability within the family and community Work with community related to: Needs of individuals with disability Potential role and contribution of individual with disability Issues related to access Include individual with disability and their family in identifying and implementing solutions
Quality of life	Help individual with disability, their family, communities assume leadership roles in meeting the needs of all citizens General health and hygiene issues Access issues Recreation and leisure time activities Foster collaboration between local and regional resources to meet health care, recreation, employment opportunities for individuals with disabilities and their families

The efforts of CCI have helped build community self-sufficiency in meeting the health care and rehabilitation needs of all Ecuadorian citizens and a greater awareness of the abilities and potential contributions of individuals with disabilities. Local leadership has been developed that supports improved health conditions in local communities. This leadership has become an important part of the community and has demonstrated its resilience as some communities are faced with challenges following recent earthquakes.

The CCI Mission states: it starts with one … but it grows into something amazing: a thriving, vibrant community, a changed nation. CCI has had the good fortune to partner with strong local partners in Ecuador and a visionary government supporting improved resources and quality of life for individuals with disabilities.

## Author Contributions

Both authors (DC and ZA) fully contributed to the conception and design of the manuscript. DC drafted the work and ZA and DC critically reviewed and revised the work. Both DC and ZA gave final approval of the submitted manuscript and agreed that they are accountable for all aspects of the work related to accuracy and integrity.

## Conflict of Interest Statement

The authors declare that the community case report was conducted in the absence of any commercial or financial relationships that could be construed as a potential conflict of interest.
